# Introducing the ArsR-Regulated Arsenic Stimulon

**DOI:** 10.3389/fmicb.2021.630562

**Published:** 2021-03-03

**Authors:** Rachel Rawle, Tara C. Saley, Yoon-Suk Kang, Qian Wang, Seth Walk, Brian Bothner, Timothy R. McDermott

**Affiliations:** ^1^Department of Microbiology and Immunology, Montana State University, Bozeman, MT, United States; ^2^Department of Land Resources and Environmental Sciences, Montana State University, Bozeman, MT, United States; ^3^Department of Chemistry and Biochemistry, Montana State University, Bozeman, MT, United States

**Keywords:** ArsR, arsenite, regulation, transcriptomics, global

## Abstract

The microbial *ars* operon encodes the primary bacterial defense response to the environmental toxicant, arsenic. An important component of this operon is the *arsR* gene, which encodes ArsR, a member of the family of proteins categorized as DNA-binding transcriptional repressors. As currently documented, ArsR regulates its own expression as well as other genes in the same *ars* operon. This study examined the roles of four ArsR proteins in the well-developed model Gram-negative bacterium *Agrobacterium tumefaciens* 5A. RNASeq was used to compare and characterize gene expression profiles in ± arsenite-treated cells of the wild-type strain and in four different *arsR* mutants. We report that ArsR-controlled transcription regulation is truly global, extending well beyond the current *ars* operon model, and includes both repression as well as apparent activation effects. Many cellular functions are significantly influenced, including arsenic resistance, phosphate acquisition/metabolism, sugar transport, chemotaxis, copper tolerance, iron homeostasis, and many others. While there is evidence of some regulatory overlap, each ArsR exhibits its own regulatory profile. Furthermore, evidence of a regulatory hierarchy was observed; i.e. ArsR1 represses *arsR4*, ArsR4 activates *arsR2*, and ArsR2 represses *arsR3*. Additionally and unexpectedly, *aioB* (arsenite oxidase small subunit) expression was shown to be under partial positive control by ArsR2 and ArsR4. Summarizing, this study demonstrates the regulatory portfolio of arsenite-activated ArsR proteins and includes essentially all major cellular functions. The broad bandwidth of arsenic effects on microbial metabolism assists in explaining and understanding the full impact of arsenic in natural ecosystems, including the mammalian gut.

## Introduction

Arsenic (As) is rated by the United States Environmental Protection Agency as a priority environmental toxin because of its occurrence, toxicity, and potential for human exposure (ASTDR, 2016), and it is ranked among the top 10 environmental threats to human health (WHO, 2016). Arsenic contamination derives from natural geologic sources and anthropogenic inputs, but regardless of source, it is recognized that microorganisms are major drivers of As chemical speciation, which influences As toxicity and mobility (Inskeep et al., 2002; Rosen, 2002; Stolz et al., 2006; Kruger et al., 2013). Consequently, understanding how and why microbes react to As in their environment is important with respect to issues involving land resource management (Inskeep et al., 2002), as well as human medicine (i.e. gastrointestinal tract) (Coryell et al., 2018; McDermott et al., 2019).

A well-characterized microbial response to As involves resistance encoded by the *ars* operon, which at the minimum is composed of *arsR*, *arsB* (alternatively *acr3*), and *arsC* (Mukhopadhyay et al., 2002; Rosen, 2002; Slyemi and Bonnefoy, 2012). ArsR, encoded by *arsR*, is a DNA binding transcriptional repressor that regulates its own expression and that of the other genes in the same *ars* operon; *arsB* or *acr3* encodes active efflux that is the primary mechanism for removing arsenite [As(III)] from the cell; and *arsC* encodes an arsenate [As(V)] reductase. When As(III) enters the cell through aquaglyceroporins, it interacts with basal (non-induced) levels of ArsR in the cell, causing ArsR to undergo a conformational change that results in release from its DNA binding site and thus opening the *ars* operon for transcription. This leads to significantly increased levels of ArsB/Acr3 and ArsC that constitutes the basic As resistance response (Mukhopadhyay et al., 2002; Rosen, 2002). Many other *ars* genes with variable roles in arsenic resistance have been recently reviewed (Andres and Bertin, 2016; Fekih et al., 2018).

In addition to the *ars* operon response, the microbial response to As is considered global (Andres and Bertin, 2016), yet the regulatory basis remains to be fully elucidated. In addition to ArsR control of the *ars* operon, the PhoRB two-component phosphate stress response (PSR) system has been shown to be the master regulator of the AioSR two-component system (Wang et al., 2018) that, along with AioX (Liu et al., 2012), controls expression of As(III) oxidase. We have also just recently reported how AioS regulatory controls extend beyond As(III) oxidation *per se* (Rawle et al., 2019). It is important to distinguish the ArsR-based response from the PhoRB–AioSR regulatory network that is induced when the cell senses low phosphate in its environment in addition to sensing As(III). This contrasts with ArsR-based control, which only requires As(III) and is insensitive to environmental phosphate.

*Agrobacterium tumefaciens* strain 5A carries two recognizable *ars* loci [Supplementary Figure 1, previously published by Rawle et al. (2019), but provided here for reader convenience], with two *arsR* genes located at each (Kang et al., 2016). These loci/genes in strain 5A have been a focal point in our past reports (Kang et al., 2012, 2015, 2016; Rawle et al., 2019) and continue to be of particular interest in this study because of significant regulatory and functional activity encoded by key genes that are of importance to arsenic-linked gene transcriptional regulation, arsenite oxidation, and phosphorus acquisition. ArsR1 and ArsR4 share 93% identity and 96% similarity, but are different from ArsR2 and ArsR3, which are more closely related to each other (78% identity and 86% similarity) (Kang et al., 2016). ArsR1 represses the nearby *phoB-1* and *pstS-1* genes (Kang et al., 2016) that are involved in the cell response to As(III), implying that the ArsR regulatory roles extend beyond that suggested by the decades of literature regarding ArsR function (Rosen, 2002). To further examine this, the current study utilized RNASeq to assess the regulatory bandwidth of each of the four characterized ArsR proteins in this bacterium. Wild-type cells were compared to isogenic *arsR* mutants grown under high phosphate culture conditions so as to distinguish the regulatory roles of these ArsRs from the PhoRB-AioXSR controls. In contrast to the current paradigm that describes ArsR proteins as only being repressors of *ars* operons, this study shows that these proteins are involved in regulating an extensive range of cell functions, and as such assists in explaining the regulatory underpinnings of bacterial global As responses.

## Materials and Methods

### Strains and Plasmids

Previously constructed in-frame deletion *arsR* mutants and *lacZ* reporter constructs in pLSP-KT2lacZ (Kang et al., 2012) were used for this study (Supplementary Table 1). β-Galactosidase reporter assays were conducted as previously described (Kang et al., 2012, 2016); however, prior efforts showed that an *arsR3:lacZ* construct does not up-regulate in response to As(III) (Kang et al., 2016) and so consequently no reporter assays were conducted for this gene. A *lacZ*:*aioB* construct was also used for *aioB* induction assays, and its construction was also as previously described (Kang et al., 2012).

### Bacterial Growth Conditions

*Agrobacterium tumefaciens* strain 5A wild-type and Δ*arsR* mutants were maintained on minimal mannitol (MMNH_4_) agar or cultured in liquid MMNH_4_ (Somerville and Kahn, 1983). Reporter constructs were maintained by adding 500 μg/ml kanamycin. For RNASeq work, cultures were grown and incubated in liquid MMNH_4_ at 30°C in water bath incubators with aeration by shaking. As(III) was added at the concentrations indicated to initiate inductions. The growth of bacterial cultures was monitored as culture optical density by a SpectraMax microtiter plate reader (Molecular Devices, California). For these experiments, the MMNH_4_ was modified to contain 10-fold reduced iron to accommodate RNA yields. Normal MMNH_4_ iron levels (62 μM) were found to interfere with the RNA extraction and purification kit protocol, reducing RNA yields to unworkable levels. Reducing initial Fe^3+^ content to 6.2 μM for the 40-min inductions at the cell densities used does not constitute an iron starvation scenario (Hamza et al., 1998; Small et al., 2009; Strange et al., 2011), which would otherwise require Fe-specific chelators (e.g., 2,2′-dipyridyl) (Bollinger et al., 2001). Further, in the current study, transcription of genes encoding iron acquisition/metabolism functions in the wild-type strain displayed patterns that were roughly balanced between up-regulation and down-regulation at the highest As(III) or were specific to a particular ArsR protein in the mutant analyses. We interpreted this as meaning that under the experimental short induction conditions employed, the cells were responding to As(III) and not activating an iron starvation response.

### RNA Extraction and Purification

Overnight cultures were diluted to OD_595_ = 0.1 and allowed to grow to an OD_595_ of 0.2 to ensure cells were in the growth phase when As(III) was added. Each treatment was incubated at 30°C in a water bath incubator aerated by shaking with the appropriate As(III) concentration for 40 min. Cells were collected by centrifugation (8000 × *g* for 5 min at 4°C), resuspended in 1 ml of Qiagen RNAprotect to protect cellular RNA and then centrifuged at 5000 × *g* at 4°C for 10 min. Cell pellets were suspended by repeated gentle pipetting in 200 μl of cold TE buffer (10 mM Tris–HCl and 1 mM EDTA, pH 8.0) containing 1 mg/ml lysozyme. RNA was then extracted using the Qiagen RNeasy Minikit following the manufacturer’s protocol, including on-column DNase digestion. RNA concentrations were measured with the SpectraMax microtiter plate reader, and RNA quality and purity were determined with an Agilent 2100 Bioanalyzer (Agilent Technologies). RNA was stored at −80°C until sequencing.

### RNA Sequencing

Sequencing was completed at Brigham Young University (Provo, UT, United States). Briefly, ribosomal RNA was removed using the Illumina Ribo-Zero rRNA removal kit for Gram-negative bacteria. The resulting RNA was prepared for sequencing using the Illumina TruSeq Stranded Total RNA Sample Prep and fragmented into 50-bp segments and reverse transcribed into cDNA. Sequence adapters used to initiate DNA polymerase binding were ligated to the cDNA, and the sequences were amplified with PCR. The cDNA library was sequenced in high output mode using Illumina sequencing technology (Illumina HiSeq 2500 sequencing platform), generating ∼12 million reads per sample. All sequence reads have been submitted to Genbank and can be found as BioSample accession: SAMN10686493, BioProject ID: PRJNA512538.

### Sequence Analysis

BowTie (Langmead and Salzberg, 2012) was used to remove any remaining ribosomal RNA sequences. Quality control was completed with FASTQC (Version 0.11.9; Andrews, 2014) and adaptors trimmed with Trimmomatic (Version 0.36; Bolger et al., 2014). mRNA sequences were aligned and quantified using Kallisto (Version 0.43; Bray et al., 2016), including 100 bootstraps for assessment of technical variance. Parameters for alignment were as follows: kmer size of 31; sequence length 180 and standard deviation of 20; and the Ensembl Bacteria genome for *A. tumefaciens* 5A (ASM23612v2) (Kersey et al., 2018). The R package Sleuth (Pimentel et al., 2017) was used to perform differential gene expression analysis using the bootstraps performed in Kallisto to adjust for technical variance. Groups were compared using the Wald Test, and *p* values were adjusted for multiple comparisons using the Benjamini and Hochberg method (Benjamini and Hochberg, 1995). Reads were normalized to transcripts per kilobase million (TPM) by dividing transcript number by gene length (in kilobases), and then reads per kilobase were counted within a sample and that number was divided by 1 million for a per-million scaling factor. Only differentially expressed genes with TPM > 1, a fold change greater than ± 2, and a *q* value ≤ 0.05 were included in the analysis. There were numerous instances where expression changes were statistically significant but failed to reach the fold change criterion and sometimes observed for genes within (apparent) operons in which other genes changed expression by > 2.0. Gene IDs were converted using UniProt (Consortium, 2019) to access available E.C. numbers, gene ontology terms, and gene names. Transcript names from Sleuth were input to UniProt for gene annotation purposes, and any genes annotated as encoding “unidentified proteins” were individually BLAST searched (Coordinators, 2016). The assignment of the function category was also based on UniProt annotation.

### Motif Analysis

The MEME suite of tools (Bailey et al., 2009) was used for identifying DNA sequences as putative ArsR1 binding sites to facilitate repression. Initial searches identified putative binding motifs in a DNA region spanning the *pstS1* and *phoB1* genes (Supplementary Figure 1) wherein we previously used electrophoretic mobility shift assays to show strong evidence of at least two ArsR1 binding sites (Kang et al., 2016). These motifs were then used as queries to scan the upstream 300 nucleotides of genes (i.e. an area similar in size to the *pstS1*–*phoB1* region) that exhibited transcriptional patterns consistent with them being transcriptionally repressed by ArsR1. This was done using the Find Individual Motif Occurrence algorithm in MEME Suite (5.0.1). FIMO utilizes log-posterior odds scoring and position-specific priors to search for a given motif (Grant et al., 2011; Cuellar-Partida et al., 2012). Motif search and queries were conducted using default settings and a *p* value ≤ 0.001 indicating a significant match. Representative sequence logos were constructed using WebLogo (2.8.2, Berkeley, CA, United States).

## Results

### Induction Optimization

**FIGURE 1:**
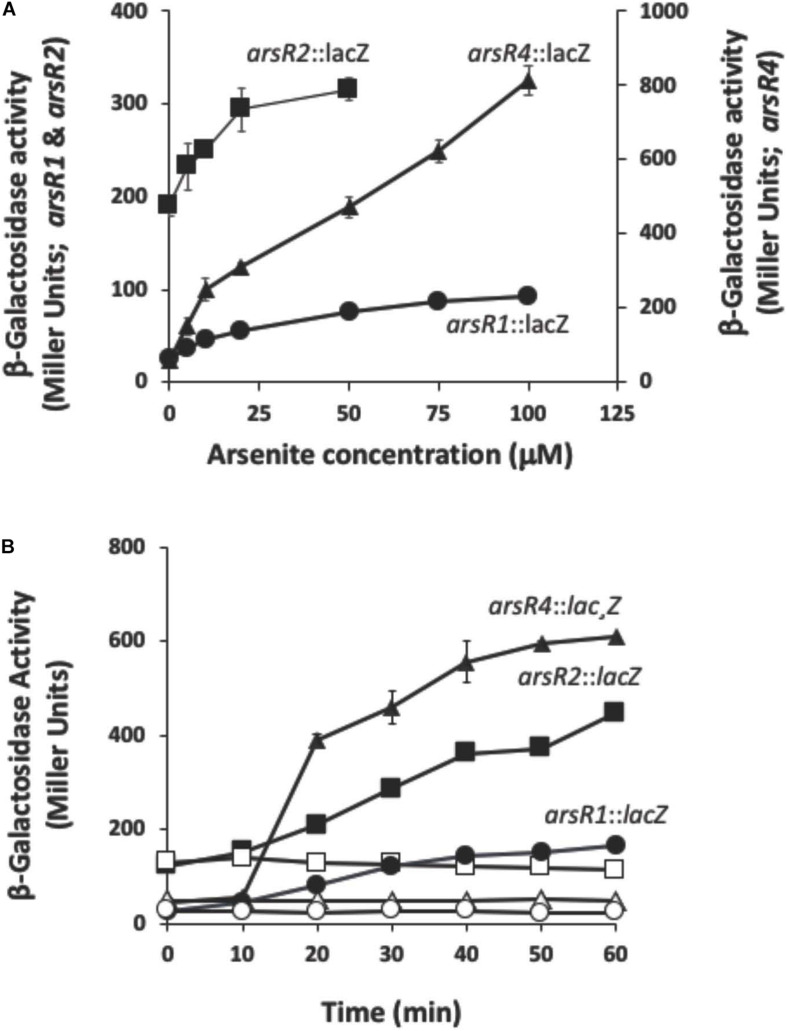
Reporter gene assays monitoring expression of *arsRl, arsR2*, and *arsR4* as a function of As(lll) exposure and time. **(A)** Reporter gene assay monitoring expression as a function of As(lll) exposure. **(B)** Reporter gene assay monitoring expression as a function of time. Filled symbols, +As(lll); open symbols, –As(lll) controls. Where visible, error bars indicate ± 1 standard deviation.

*arsR* β-galactosidase reporter constructs were used to optimize As(III) concentration and induction time for each *arsR.* These data were then used for characterizing their regulatory activities by RNASeq (below). Each reporter had a distinct profile and level of expression. The non-induced (constitutive) expression levels ranged from 26 Miller units (MU) for *arsR1*:*lacZ* to 190 MU for *arsR2*:*lacZ* (Figure 1A). Reporter response varied (Figure 1A), with *arsR4*:*lacZ* showing the largest response relative to the other reporters. Other assays examined optimal induction times (Figure 1B). After considering reporter responses in both assays, As(III) concentrations selected for the induction treatment were 100 μM for *arsR1*:*lacZ*, 50 μM for *arsR2*:*lacZ*, and 75 μM for *arsR4*:*lacZ* and induction periods of 40 min. By limiting the As(III) exposure period, the goal was to induce an As(III) transcriptional response but constrain/avoid potential secondary wave(s) of transcriptional responses from other potential transcriptional regulators that might be controlled by these ArsRs. Further, extending incubation times did not appreciably enhance *arsR* reporter expression levels. Since ArsR proteins are typically viewed to autoregulate their own gene, we inferred that optimized induction of these *arsR* genes would also reflect near-optimal As(III)-triggered release of these ArsR from other similar DNA binding sites in the genome.

### Wild-Type Gene Transcription Changes

Based on the reporter gene assays, initial RNASeq experiments compared the response of −As(III) and +As(III) wild-type *A. tumefaciens* cells exposed to 50, 75, and 100 μM As(III) for a 40-min incubation period. A total of 138 genes were up-regulated and 111 genes were down-regulated in the wild-type strain exposed to As(III). Table 1 emphasizes selected functions that were represented by multiple genes (i.e. suggests non-random, organized, cell-wide response) as well as genes/operons depicted in Supplementary Figure 1 (see Supplementary Table 2 for a complete list of genes with UniProt gene identifiers). For reader convenience, expression data in Table 1 also include expression data for each of the four *arsR* mutants to facilitate a direct contrast to the wild-type strain and will be further discussed as total responses below. As expected based on prior research of numerous organisms (Andres and Bertin, 2016; Fekih et al., 2018) and the reporter assays (Figure 1), *ars* genes involved in As resistance showed increased expression in response to As(III) (Table 1). Up-regulation of the *ars* genes was consistent with what we have previously documented for strain 5A (Kang et al., 2012, 2016) using an orthogonal technique (*lacZ* reporters and reverse transcriptase PCR for a subset of the genes shown in Supplementary Figure 1) (Kang et al., 2012, 2016). Since these RNASeq-based *ars* gene transcriptional responses were observed in the same batches of mRNA used in the RNASeq libraries for all other transcriptional responses, they serve as internal controls to validate the entire data set. Except for *aioB* (see below), the *aio* genes [involved in As(III) oxidation] were not up-regulated because the cultures were incubated in high phosphate media, which inhibits their induction (Kang et al., 2012; Wang et al., 2018). This is consistent with prior work and thus also provides validation.

**TABLE 1:** Gene expression profiles of selected major functional groups in the wild type strain 5A and the four *arsR* mutant derivatives as a function of As(III) exposure.

		Wild type			Δ*arsR1*		Δ*arsR2*		Δ*arsR3*		Δ*arsR4*	
Uniprot identifier	Annotation	50 μM	75 μM	100 μM	0	100 μM	0	50 μM	0	75 μM	0	75 μM
***Arsenic resistance/metabolism***												
AT5A_08115	Transcriptional regulator, ArsR family			2.1								
AT5A_22226	ArsR3 transcriptional regulator			4.4			2.5					
AT5A_22236	ArsH2 Arsenical resistance protein	23.3	35.3	92.7	**4.5**							
AT5A_22241	ArsC3 Arsenate reductase	31.2	45.0	124.1	**4.2**							
AT5A_22246	Acr3-2 Arsenite efflux pump protein	44.7	63.9	178.8	**5.9**						2.4	
AT5A_22251	ArsR4 transcriptional regulator	34.7	51.9	135.5	**5.9**							
AT5A_25245	ArsR family transcriptional regulator	2.5	3.2	18.1			3.3					
AT5A_25620	ArsC Arsenate reductase	11.7	13.7	16.2	6.5							
AT5A_25645	ArsR1 transcriptional regulator	43.7	52.4	60.8								
AT5A_25650	ArsC4 Arsenate reductase	35.2	43.8	61.3	12.4							
AT5A_25655	ArsC1 Arsenate reductase	66.7	89.5	150.4	13.3							
AT5A_25660	Acr3-1 Arsenite efflux pump	60.5	82.8	126.0	10.4							
AT5A_25665	ArsC-2 Arsenate reductase	53.9	74.6	122.3	10.2							
AT5A_25670	ArsH1 Arsenical resistance protein	50.3	70.2	104.1	8.9							
AT5A_25685	ArsR2 transcriptional regulator		2.1	7.0							−2.0	
AT5A_25560	*aioA*, arsenite oxidase large subunit									2.1		
AT5A_25565	*aioB*, arsenite oxidase small subunit (aioB)				−4.1	−2.1	−3.7	−2.0	−3.4		−3.6	

***Chemotaxis***		**Wild type**			**Δ*arsR1***		**Δ*arsR2***		**Δ*arsR3***		**Δ*arsR4***	
**Uniprot identifier**	**Annotation**	**50 μM**	**75 μM**	**100 μM**	**0**	**100 μM**	**0**	**50 μM**	**0**	**75 μM**	**0**	**75 μM**

AT5A_02480	Methyl-accepting chemotaxis protein							−2.3				
AT5A_10140	CheB Chemotaxis response regulator protein			−2.7			−2.7	−2.8			−2.0	
AT5A_10150	CheW Chemotaxis protein			−2.1								
AT5A_10155	Methyl-accepting chemotaxis protein			−2.1								
AT5A_10160	CheW Chemotaxis protein			−2.2								−2.4
AT5A_10165	Chemotaxis protein histidine kinase			−2.5			−2.0					−2.5
AT5A_10170	Chemotaxis receiver protein			−2.2								−2.3
AT5A_12222	Methyl-accepting chemotaxis protein			−2.1			−2.0					
AT5A_12862	Methyl-accepting chemotaxis protein A						−2.5					
AT5A_24110	Methyl-accepting chemotaxis protein						−2.4					

***Copper metabolism/tolerance***		**Wild type**			**Δ*arsR1***		**Δ*arsR2***		**Δ*arsR3***		**Δ*arsR4***	
**Uniprot identifier**	**Annotation**	**50 μM**	**75 μM**	**100 μM**	**0**	**100 μM**	**0**	**50 μM**	**0**	**75 μM**	**0**	**75 μM**

AT5A_04590	Transcriptional regulator, MerR family	-2.9		−3.7	−4.2	−2.1	−4.1		−2.6			
AT5A_22416	Uncharacterized protein	-5.9	−5.8	−13.9	−37.5				−2.5		−13	
AT5A_22421	Cu tolerance protein	-5.5	−5.1	−13.5	−38.5				−2.8		−11	
AT5A_22426	Multicopper oxidase	-3.3	−3.2	−8.7	−33.4		−4.5		−2.5		−4.9	
AT5A_22431	Cu tolerance protein	-2.3	−2.4	−8.1	−26.3				−2.2		−3.5	
AT5A_22441	Uncharacterized protein associated with apparent Cu tolerance operon				−5.1		−2.4				−2.6	

***Iron acquisition***		**Wild type**			**Δ*arsR1***		**Δ*arsR2***		**Δ*arsR3***		**Δ*arsR4***	
**Uniprot identifier**	**Annotation**	**50 μM**	**75 μM**	**100 μM**	**0**	**100 μM**	**0**	**50 μM**	**0**	**75 μM**	**0**	**75 μM**

AT5A_00040	Periplasmic chelated iron-binding protein			5.0		−3.4						2.7
AT5A_00050	Iron ABC transporter ATP-binding protein			3.4		−2.4						
AT5A_01140	Iron ABC transporter nucleotide binding			−2.8								
AT5A_01170	Iron ABC transporter nucleotide binding/ATPase					2.1	2.7					
AT5A_01175	Iron ABC transporter membrane spanning					2.5	4.0					
AT5A_01180	Iron ABC transporter substrate binding protein				2.5		6.0					
AT5A_05975	Fe III dicitrate ABC transporter, permease					3.0						
AT5A_05990	Ferritin								2.4			
AT5A_08610	TonB-dependent heme receptor A								7.7			
AT5A_09670	Iron-chelator utilization protein							−2.6				
AT5A_09690	HmuV Hemin import ATP-binding protein			−3.8								
AT5A_09695	Hemin ABC transporter transmembrane protein			−6.7								
AT5A_10100	FbpC Fe(3+) ions import ATP-binding protein			2.4		−5.1	4.6				2.0	
AT5A_10110	Iron ABC transporter substrate-binding protein	-3.2	−3.4	3.3	−2.3	−7.7	8.8		−2.3			
AT5A_10115	Iron ABC transporter substrate-binding protein					−2.5	3.4					
AT5A_10942	Iron ABC transporter substrate-binding protein							−2.5				
AT5A_11337	Iron-regulated protein			−3.3								
AT5A_11917	Iron ABC transporter nucleotide-binding			−2.7								
AT5A_11927	Iron ABC transporter transmembrane protein			−2.1								
AT5A_15846	Iron ABC transporter, membrane spanning			−2.3	−2.2		−2.4					
AT5A_16421	Ferrichrome ABC transporter			−2.2				−2.1				
AT5A_16431	Ferrichrome transport system permease			−2.6				−2.1				

***Iron acquisition***		**Wild type**			**Δ*arsR1***		**Δ*arsR2***		**Δ*arsR3***		**Δ*arsR4***	
**Uniprot identifier**	**Annotation**	**50 μM**	**75 μM**	**100 μM**	**0**	**100 μM**	**0**	**50 μM**	**0**	**75 μM**	**0**	**75 μM**

AT5A_16436	Ferrichrome ABC transporter							−2.5				
AT5A_16631	Fe3+ siderophore ABC transporter permease			−2.4								
AT5A_16636	Fe3+ siderophore ABC transporter permease							−2.4				
AT5A_23490	TonB-dependent siderophore receptor							−2.3	5.5			
AT5A_23515	Iron ABC transporter permease			−2.6				−2.2				
AT5A_23520	Iron ABC transporter periplasmic							−2.3				
AT5A_23560	2,3-dihydro-2,3-dihydroxybenzoate synthetase							−2.6				
AT5A_23725	Iron ABC transporter permease	2.0	2.0									

***Phosphate stress response***		**Wild type**			**Δ*arsR1***		**Δ*arsR2***		**Δ*arsR3***		**Δ*arsR4***	
**Uniprot identifier**	**Annotation**	**50 μM**	**75 μM**	**100 μM**	**0**	**100 μM**	**0**	**50 μM**	**0**	**75 μM**	**0**	**75 μM**

AT5A_10832	PhnH Carbon-phosphorus lyase complex subunit			2.1								
AT5A_11977	PstS2 Phosphate ABC transporter substrate-binding			2.3		−2.3						
AT5A_25585	PhoB1 Phosphate regulon regulatory protein	9.0	9.8	14.7	4.8							
AT5A_25590	PstS1 Phosphate binding	8.6	10.1	14.1	5.0							
AT5A_25595	PstC1 Phosphate transport system permease	3.0	3.7	3.9	1.9							
AT5A_25600	PstA1 Phosphate transport permease protein	6.5	8.3	8.2	3.3							
AT5A_25605	PstB1 Phosphate import ATP-binding protein	4.3	5.2	6.5	3.0							
AT5A_25610	PhoU1 Phosphate transport system protein	3.9	4.8	5.5	2.4							
AT5A_25615	Phosphate regulon regulatory protein	5.7	7.1	7.9	2.2							
AT5A_25625	Phosphonates import ATP-binding	7.7	7.9	15.6	6.0							
AT5A_25630	PhnC Phosphonate ABC transporter	6.6	6.9	8.7	3.8							
AT5A_25635	Phosphonate ABC transporter, inner membrane	3.9	4.2	4.8	2.7							
AT5A_25640	Phosphonate ABC transporter, inner membrane	3.1	2.8	3.4								

***Sugar transporters***		**Wild type**			**Δ*arsR1***		**Δ*arsR2***		**Δ*arsR3***		**Δ*arsR4***	
**Uniprot identifier**	**Annotation**	**50 μM**	**75 μM**	**100 μM**	**0**	**100 μM**	**0**	**50 μM**	**0**	**75 μM**	**0**	**75 μM**

AT5A_00215	Ribose ABC transporter substrate binding		2.3		2.2		3.1		2.6			
AT5A_00220	Ribose ABC transporter nucleotide binding		2.0				2.4		2.1			
AT5A_01015	Monosaccharide-transporting ATPase			2.3								
AT5A_03235	Putative multiple sugar transport system						2.3					
AT5A_03250	Sugar ABC transporter permease						2.1					
AT5A_03255	Sugar ABC transporter nucleotide-binding						2.4					
AT5A_03260	Sugar ABC transporter ATPase						2.6					
AT5A_09935	Sugar ABC transporter substrate-binding protein						2.6					
AT5A_09940	Sugar ABC transporter transmembrane protein						2.3					
AT5A_09945	Sugar ABC transporter transmembrane protein						2.1					
AT5A_09950	Sugar ABC transporter nucleotide-binding ATPase						2.2					
AT5A_18906	Maltose ABC transporter transmembrane protein											−2.4
AT5A_18911	Maltose ABC transporter transmembrane protein											−2.4
AT5A_18916	Maltose ABC transporter substrate-binding protein											−2.7
AT5A_18921	Maltose/maltodextrin ABC transporter											−2.7
AT5A_19366	Sugar ABC transporter periplasmic sugar-binding protein	7.2	7.8	−5.5	5.7	26.5	7.0		5.9		5.1	
AT5A_19371	Sugar ABC transporter permease	5.7	6.3	−6.1	4.3	25.4	5.4		4.7		4.0	
AT5A_19376	Multiple sugar transport system permease protein	4.2	4.6	−9.1	3.1	30.9	3.7		3.5		3.0	
AT5A_19386	Sugar ABC transporter ATP-binding protein	2.2	2.5	−17.2		33.6	2.1					−2.0
AT5A_19781	Ribose ABC transporter transmembrane protein											
AT5A_22281	Sugar ABC transporter permease						2.4					
AT5A_22286	Sugar ABC transporter substrate-binding protein						2.4					
AT5A_24025	Sugar ABC transporter substrate-binding protein						2.3		2.6		2.2	
AT5A_24035	Sugar ABC transporter nucleotide ATPase						2.0		2.6		2.2	
AT5A_24045	Sugar ABC transporter permease						2.0		2.9		2.1	
AT5A_24050	Sugar ABC transporter permease								2.40			

Gray highlighted data identifies genes uniquely affected in the respective ΔarsR mutant and presumed to be controlled directly or indirectly by only that ArsR. Under the “Wild type” columns, all FC comparisions are wild type with As(III):wild type without As(III). For each of the arsR mutants, “0” indicates the mutant: wild type and the “100 μM, 50 μM, and 75 μM” indicate their respective mutant with As(III):wild type with equivalent As(III) exposure. For -As(III) treatments of mutants, positive changes in gene expression illustrate genes we view to be normally repressed (directly or indirectly) by the respective ArsR, whereas negative changes in expression infer genes normally activated (directly or indirectly) by the respective ArsR. Red text identifies arsR genes. * and green highlighted Uniprot identifier numbers denotes that the grouped genes are either in a known operon or are physically associated and oriented so as to suggest they are co-expressed as an operon. Areas with no FC indicate that under that scenario, the expression changes were between -2 and 2 and/or not significant. Only significant expression changes with a fold change greater than two are shown; **blue bold text** denotes changes in expression in the ars2 locus but with q-values of 0.06-0.11.

The group of validation genes includes *pst*/*pho*/*phn1* that encode aspects of phosphate and phosphonate uptake/transport and are proximal to the *ars1* locus (Supplementary Figure 1). These genes were up-regulated as observed previously for *pstS1* and *phoB1* (Kang et al., 2012, 2016), although, by contrast, no changes were observed for the separate *pho*/*pst2* locus that controls the formal PSR and is not responsive to As(III) (results not shown). Other affected functional categories include the up-regulation of genes involved in DNA replication (Supplementary Table 2), although these responses occurred primarily at the highest level of As(III) applied (100 μM). Several genes annotated as membrane proteins were also affected, including numerous ABC-type transporters (Supplementary Table 2). For genes up-regulated by the lowest As(III) level, transcription levels often increased with increasing As(III) exposure levels; e.g., all *ars* genes (As resistance) and more subtly for many *pst*/*pho*/*phn1* locus genes (Table 1 and Supplementary Table 2).

Many genes/functions were down-regulated in As(III)-exposed cells. However, this primarily occurred at the highest As(III) level, 100 μM (Table 1 and Supplementary Table 3). Interesting examples include copper metabolism/tolerance and chemotaxis (Table 1). Many iron transport/regulation genes were primarily down-regulated at the highest As(III) level as well (Table 1). We interpreted this to mean that the cells were not iron-stressed in the 40-min inductions, but rather that As(III) exposure interacts with iron homeostasis functions (see more below, *arsR3* mutant). Other down-regulated functions include membrane proteins, β-lactam resistance (five genes), transcription regulation (four genes), and molybdenum transport (four genes, Supplementary Table 2). There were also instances of As(III) concentration having reversible effects; e.g., one sugar transporter operon is up-regulated at the lower As(III) levels, but then repressed at the highest As(III) level (Supplementary Table 2, rows 326–329).

### Altered Gene Expression in the *arsR* Deletion Mutants

The next phase of analysis assessed regulatory control by the different ArsRs. Each mutant was compared to wild type. The mutant transcriptomics experiments were optimized for As(III) concentrations and exposure times as determined above (Figure 1). Since the *arsR3* reporter does not respond to As(III), 75 μM As(III) was selected as an average used for the other *arsR* genes when characterizing its regulatory profile.

**FIGURE 2:**
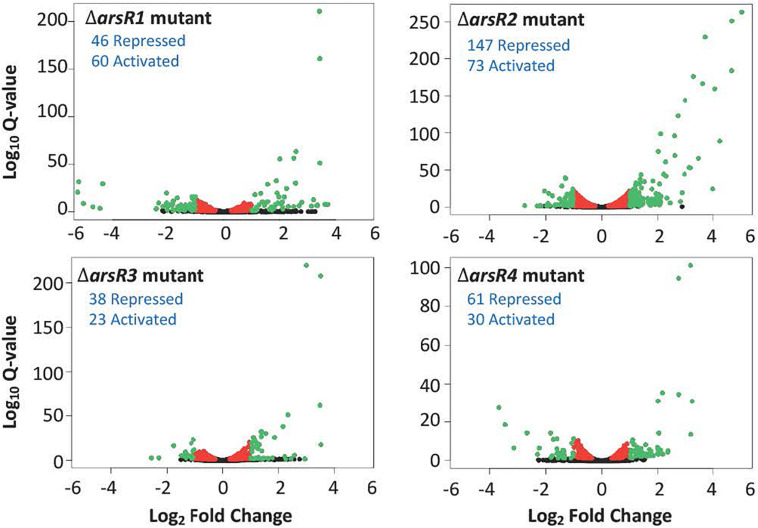
Volcano plots illustrating the influence of each ArsR on genome-wide transcription in the absence of As(lll). In each case, expression changes are calculated as the ratio of the mutant compared to the wild type. Red colored dots indicate genes that were significantly changed (*q*-value < 0.05), but less than two-fold. Green dots indicate genes that were significantly changed (*q*-value < 0.05) and had a fold change > + 2.0. Blue text indicates the number of genes with increased or decreased expression and are presumed to be activated either directly or indirectly by the respective ArsR in the wild type cell.

Based on current models of how ArsR-type repressors control gene expression, removing the repressor proteins (i.e. deletion mutations as in the current study) should result in constitutive expression of genes that are normally repressed by that protein in the absence of the de-repressing ligand [As(III) in this instance]. In the context of this study where As(III) was not added, increased transcription in the mutant relative to the wild type would indicate that the gene is normally repressed by the specific ArsR, whereas decreased expression in the mutant suggests that the gene is activated (directly or indirectly) by the respective ArsR. Overall, there were 482 genes significantly influenced by one or more of the ArsRs in the absence of As(III). A total of 295 genes were classified as repressed and 187 genes were classified as activated, spanning a broad range of cell physiology. The volcano plots (Figure 2) offer a comparative visual assessment of total gene expression patterns for each mutant relative to the wild type in the absence of As(III). Both positive and negative changes in gene transcription were observed for all four mutants, with ArsR2 exhibiting patterns that suggest it has the greatest influence over cellular gene expression (220 genes total), whereas ArsR3 influences the fewest (61 genes) (Figure 2).

**FIGURE 3:**
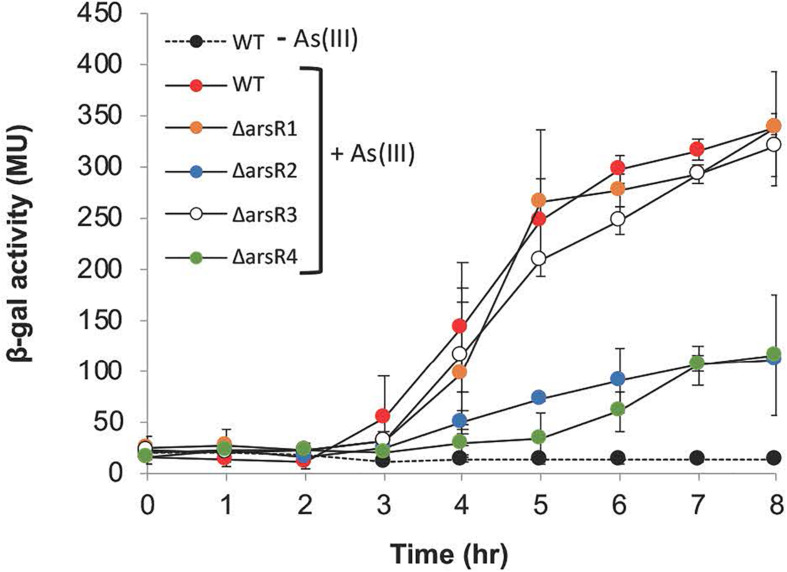
Reporter gene *(aioB:lacZ)* assays monitoring *aioB* induction as a function of *arsR* genotype, As(lll) exposure and time. Where visible, error bars indicate ± 1 standard deviation.

Surprisingly, *aioB* (AT5A_25565) expression was altered in all *arsR* mutants, and in all cases expression was reduced, indicating that these ArsrR proteins have an activating influence (Table 1). This suggests that in the wild-type strain, one or more of these ArsR proteins have some type of positive influence over *aioB* expression. This was unexpected because strong experimental evidence has shown that the *aioBA* genes are controlled by AioXSR (reviewed by Andres and Bertin, 2016). Therefore, additional experiments were conducted under full induction potential conditions [i.e. low phosphate plus 100 μM As(III)] to determine if any of these ArsRs exert control over *aioB*. As can be seen in Figure 3 and as we have reported previously (Kashyap et al., 2006; Kang et al., 2012), the *aioB*:*lacZ* reporter in the wild-type strain failed to induce in the absence of As(III); however, it was induced at the expected times and levels when the cells were incubated with As(III). The same induction profiles were observed in the *arsR1* and *arsR3* mutants; however, full induction was not achieved in the *arsR2* and *arsR4* mutants, demonstrating ArsR2 and ArsR4 are involved at some level in positively regulating *aioB* (Figure 3 and Table 1).

### Specific Functions Associated With Individual ArsRs

Table 1 highlights mutant:wild-type comparisons with respect to what appears to be major, coordinated transcriptional changes for several cellular functions. Comprehensive lists for each *arsR* mutant are provided in Supplementary Tables 3–6, and demonstrate that virtually every aspect of cell physiology is influenced. ArsR1 appears to be the major repressor of *ars1* arsenic resistance locus/operon (Supplementary Table 3) and the PSR related activities encoded by the *pho/pst1* operon (Supplementary Figure 1), which is physically located near the *ars1* locus (Supplementary Figure 1). ArsR1 control over the *pst/pho/phn1* locus (Table 1) is consistent with our prior DNA binding experiments, which showed that purified ArsR1 would bind at two distinct locations between *pstS1* and *phoB1* (Kang et al., 2012) (please refer to Supplementary Figure 1). Expression changes of genes in the distal *ars2* locus, including *arsR4*, also reflect repression by ArsR1 (Supplementary Table 3). Additionally, ArsR1 appears to be involved at some level in activating (directly or indirectly) functions such as amino acid metabolism, copper tolerance, iron acquisition, nickel transport, pilus assembly/Type VI secretion, and succinoglycan biosynthesis (Supplementary Table 3).

ArsR2 exhibits repressor activity controlling cobalamin synthesis, heat/cold shock responses, iron acquisition, and six different sugar transport operons. Additionally, it appears to be a repressor of *arsR3*. Indeed, some of the genes apparently activated by ArsR2 are also activated by ArsR3 (compare Supplementary Tables 4, 5). ArsR2 regulatory control of *ars* genes is greatly reduced relative to ArsR1, but ArsR2 does exclusively control the uncharacterized *arsR* gene (AT5A_25245). This demonstrates regulatory interaction between these ArsR proteins, as was observed for ArsR1 exerting control over *arsR4* (Table 1). Other genes up-regulated in the Δ*arsR2* mutant (presumed repressed in wild type) encode transporters for oligonucleotides, amino acids, iron, and six disparately located ABC-type sugar transporters (Supplementary Table 4). Functions apparently activated by ArsR2 in the absence of As(III) include various other transport activities (amino acid, nitrogen, iron, and sulfate), copper tolerance, chemotaxis, queuosine biosynthesis, purine metabolism, membrane proteins, amino acid biosynthesis, synthesis of ribosomal proteins, glycosyl transfer, and signal transduction (Supplementary Table 4).

Transcript profile comparisons showed that loss of ArsR3 was associated with the fewest changes; 38 repressed and 23 activated (Table 1 and Supplementary Table 5). Functions apparently normally repressed by ArsR3 involve nucleotide triphosphate metabolism and transport of different sugars (Supplementary Table 5). Cell activities (in)directly activated by ArsR3 include benzoate degradation, copper tolerance, and chemotaxis. Of particular note, we draw attention to the very large number of iron acquisition genes down-regulated in the *arsR3* mutant exposed to As(III); 34 genes in total, including seven full operons (Supplementary Table 5).

A comparison of the transcriptional patterns of the *arsR4* mutant indicated that expression of 91 genes was altered (Supplementary Table 6). Sixty-one showed increased expression, suggesting that they are normally repressed by *arsR4*. This includes *acr3-2* directly downstream from *arsR4*. The profile of regulated genes was similar to the other ArsR proteins, with the exception of universal stress response and a Fix operon associated with N_2_ fixation (Supplementary Table 6). It is worth noting that expression of *arsR2* decreased, indicating that ArsR4 activates ArsR2, either directly or via a secondary response. Further, chemotaxis and copper tolerance are a regulatory target for ArsR4, again as an activator as well as for a large number of genes/operons encoding aspects of iron acquisition or iron-related regulatory activity (e.g., FecR).

### Genes Regulated by Multiple ArsRs

There are numerous instances of the same gene (or apparent operon) being influenced in two or more *arsR* mutants. Examples here include evidence of (i) Acr3-2 arsenite efflux pump being primarily repressed by ArsR1, but ArsR4 also influences expression; (ii) the arsenite oxidase small subunit (*aioB*) expression was decreased in all four ArsR mutants; (iii) expression of several chemotaxis genes activated by both ArsR2 and ArsR4; and (iv) several genes involved in copper resistance/metabolism being apparently activated by three different ArsRs in the absence of As(III), with the influence by ArsR1 being strongest (Table 1).

### Mutant Responses to As(III)

The next step was to analyze the response of the *arsR* mutant strains to As(III). As one would expect given the regulatory overlap described to this point, the observed patterns of gene regulation were complex. The majority of the apparent activation effects for ArsR1 were exhibited only in As(III)-treated cultures (Supplementary Table 3), whereas in contrast very few of the ArsR4-controlled genes displayed any response to As(III) (Supplementary Table 6). Overall, four basic patterns were observed. First, expression of some genes/operons increased relative to wild type in the absence of As(III) but were the same as wild type in As(III)-exposed cultures. This expression pattern is consistent with constitutive transcription in the absence of the relevant ArsR repressor leading to fully open transcription and is not affected by the ArsR ligand, As(III), *per se*. Prominent examples of this pattern include the arsenic response and *pho/pst/phn1* locus, copper tolerance genes in the *arsR1* mutant (Table 1 and Supplementary Table 3), and nearly all of the gene/operons controlled by ArsR4 (Supplementary Table 6). A second general pattern involved genes/operons exhibiting altered expression (relative to wild type) in the absence of As(III), that was changed even more in the same direction upon As(III) exposure. An example of this pattern is the sugar transporter system (AT5A_19366, AT5A_19371, AT5A_19376, AT5A_19386) in the *arsR1* mutant (Supplementary Table 3). The third pattern involved no deviation from wild type in the absence of As(III), followed by a significant change in expression with As(III). The best examples of this pattern occurred in the As(III)-treated *arsR3* mutant wherein decreased expression was observed for iron acquisition/homeostasis (33 genes) and chemotaxis/motility (11 genes) (Supplementary Table 5). This suggests that ArsR3 normally is somehow involved in the activation of these genes, but only in As(III)-exposed cells; however, we note that expression of these same genes was not significantly altered in the wild-type cultures, regardless of As(III) treatment level (Table 1 and Supplementary Table 2). Finally, a fourth pattern observed in all mutants involved genes exhibiting increased expression in the absence of As(III) and an additional increase upon As(III) exposure. This fourth pattern implies the involvement of the relevant ArsR protein, potentially working with another, weaker regulator whose expression is As(III) sensitive.

### Motif Analysis

Our prior work found that ArsR1 exerts repressive control over *pstS1* and *phoB1* (please refer to Supplementary Figure 1) and showed that purified ArsR1 binds two sites in the DNA region between these genes (Kang et al., 2012). These genes are divergently transcribed, and their ATG translational start sites are separated by 363 nucleotides. In the current study, this region was examined to identify potential ArsR1 binding site motifs and then used these motifs as queries to scan the DNA upstream of each gene exhibiting transcriptional behavior consistent with being repressed by ArsR1 (identified in Table 1 and Supplementary Table 3). MEME software (Bailey et al., 2009) identified three distinct motifs that vary between 9 and 29 bp in length and in their degeneracy (Supplementary Figure 2). The two more conserved motifs occurred twice in the 363-nucleotide region whereas the more degenerate motif was found in four locations (Supplementary Figure 2A). Using these motifs as queries resulted in a total of 40 significant matches being found associated with the upstream regions of scanned genes (Supplementary Table 3). Based on location, orientation, and prior research (Kang et al., 2012; Kang et al., 2016), it is reasonable to predict that there are 12 operons repressed by ArsR1 (accounting for 47 genes; Supplementary Table 3). We found at least one or more of these motifs located within the upstream DNA of the promoter proximal gene for nine of these operons (Supplementary Table 3). This includes *arsR1* and the *phoB1* and *pstS1* genes that we have studied extensively and shown to be under the control of ArsR1 (Kang et al., 2012; Kang et al., 2016). Alignment of the predicted motifs revealed significant conservation for the shorter predicted motifs, whereas the longer motif was much more variable with the exception of what may be key nucleotides sharing the same spacing (Supplementary Figure 2B).

## Discussion

*Agrobacterium tumefaciens* or very closely related taxa have been frequently isolated from arsenic-contaminated soils throughout the world, including Korea (Chang et al., 2010), Australia (Santini et al., 2000), China (Cai et al., 2009a), India (Sarkar et al., 2013), and the United States (Rhine et al., 2007). *A. tumefaciens* strain 5A, used in this study, came from arsenic-contaminated soils in Montana, United States (Macur et al., 2004). Strain 5A has been used as a model organism to identify the AioSR two-component signal transduction system (Kashyap et al., 2006), the periplasmic As(III) binding protein AioX (Liu et al., 2012), co-regulation of *aioBA* induction and elements of the PSR (*pst1-phoB1* locus) (Kang et al., 2012), *ars* locus comparisons (Kang et al., 2016), and PhoR regulation of the *aioSRBA* (Wang et al., 2018), and to define the PhoR and AioS regulation profiles (Rawle et al., 2019). As such, this organism can reasonably be considered an ecologically relevant model to understand and predict how bacterial metabolism and function are influenced or disrupted by As(III).

ArsR redundancy raises obvious questions regarding the specific role of each in regulating gene expression. Questions regarding *ars* gene/operon redundancy have been posed previously. Páez-Espino et al. (2015) were able to show that temperature influenced the expression of two different *ars* operons in *Pseudomonas putida* KT2440; however, we were unable to link temperature to the expression of the *ars* genes in *A. tumefaciens* 5A (Kang et al., 2016). Evolution models would argue that genetic and functional redundancy requires some level of positive selective pressure (Francino, 2005). Sorting out explanations for such apparent paradoxes, in particular regulators such as ArsR, is not easy but we took the most direct approach by mutating each *arsR* associated with the *ars1* and *ars2* loci (Supplementary Figure 1). This allowed for a systematic parsing of the role(s) each ArsR might contribute to regulation at a minimum of three levels: (i) contributing to the global response to As(III); (ii) regulation within the *ars1/pho/pst/phn1/aio* island; and (iii) cross-regulation between the *ars1* and *ars2* loci. Each ArsR is involved in controlling its own suite of genes, though there appears to be some regulatory overlap (Table 1 and Supplementary Tables 2–5), which might be particularly predicted for ArsR1 and ArsR4, which are highly related phylogenetically, sharing 93% identity and 96% similarity (Kang et al., 2016).

**FIGURE 4:**
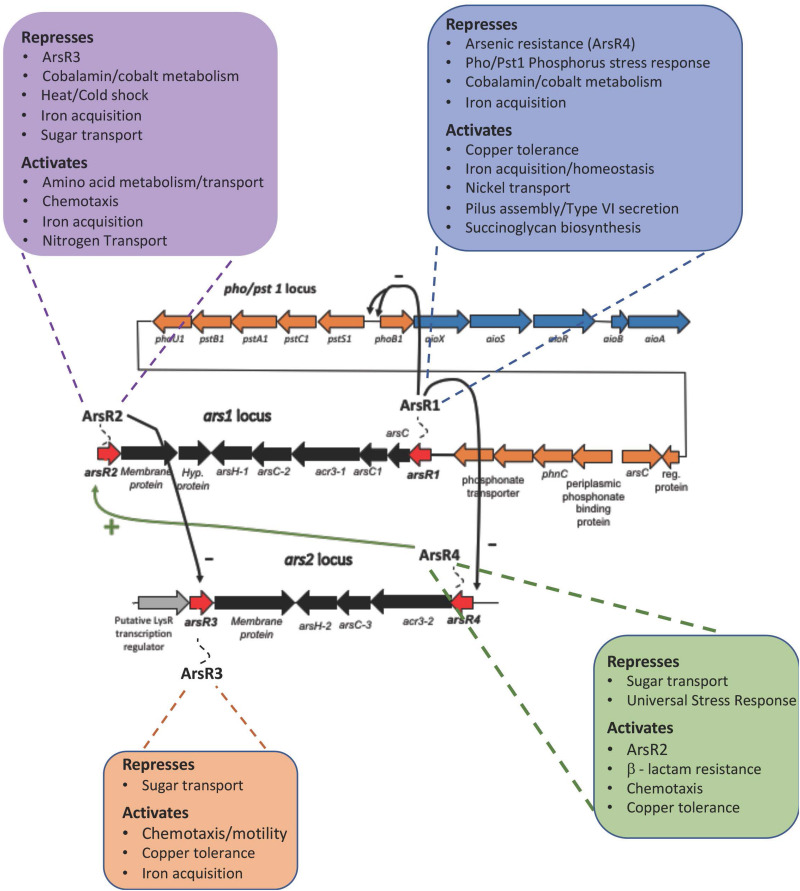
ArsR Regulatory Hierarchy. Transcription patterns are consistent with a regulatory hierarchy where ArsRl represses *arsR4*, ArsR4 activates *arsR2*, and ArsR2 represses *arsR3.* Only select, subset of functions are included in this figure to emphasize the broad bandwidth. The functions that are shown in this figure are exclusive to the ArsR protein; i.e. As response is exclusively regulated by ArsRl and displayed no changes due to ArsR2, ArsR3, and ArsR4. The “–” symbol indicates repression and the “+” symbol indicates activation.

Each of these ArsRs affects a large number of cellular functions (Table 1 and Supplementary Tables 2–5) and thus offers an initial, reasonable explanation for global As(III) responses not controlled by AioS or PhoR (see below). Noteworthy are functions represented by numerous genes or whole operons dispersed throughout the genome, implying an organized, non-random cell response. Prominent examples include regulation of copper tolerance, chemotaxis, and iron acquisition/homeostasis (Table 1 and Supplementary Tables 1, 5). Also noteworthy is that the mutant transcriptional patterns suggest that an ArsR regulatory heirarchy may be in place (Figure 4), wherein ArsR1 is a repressor of *arsR4*, ArsR4 positively influences the expression of *arsR2*, and ArsR2 represses *arsR3* (Table 1 and Figure 4).

Of the known As(III)-sensitive response systems, ArsR has been well studied by Rosen and colleagues for its role in regulating arsenic resistance encoded by the *ars* operon (Wu and Rosen, 1993; Rosen, 1995; Oremland and Stolz, 2003; Andres and Bertin, 2016). However, given the significant cellular response to As(III) (reviewed by Andres and Bertin, 2016), it was important to examine the ArsR repressor to determine the full extent of its regulatory activity. As we have clearly demonstrated here, ArsR-regulated gene expression in response to arsenic extends well beyond the current *ars* operon control paradigm. We present a new model that is considerably more complex, encompassing a wide range of cellular functions to varying degrees (Figure 4). *ars* operon redundancy has previously drawn interest (Ordóñez et al., 2005; Branco et al., 2008; Fernández et al., 2014; Páez-Espino et al., 2015; Kang et al., 2016), but functional explanations have been limited. This *ars* operon redundancy is additive for As(III) resistance (Kang et al., 2016) and temperature specificity has been shown to be relevant in *Pseudomonas putida* (Páez-Espino et al., 2015), although not in *A. tumefaciens* 5A (Kang et al., 2016).

Motif searches were conducted over a relatively broad span of 300 nucleotides upstream from the annotated translational start sites to determine if the ArsR1-regulated genes shared commonality in regard to potential targets for transcriptional regulatory elements. Query sequences for these searches were based on potential motifs identified within the *pstS1*–*phoB1* spanning region wherein we previously identified at least two ArsR1 binding sites using electrophoretic mobility shift assays (Kang et al., 2012). We note that with the exception of the TCGTCG–TGTC motif, which was near the promoter regions of both *pstS1* and *phoB1*, none of these motifs were located within a region closely associated with what would be reasonably predicted to be the -10/-35 region for these gene/operons; most were at least 100–150 nucleotides upstream. While there was significant sequence conservation for the two smaller motifs, none of these motifs correspond to the imperfect 12-2-12 inverted repeats model (Busenlehner et al., 2003; Supplementary Figure 2).

Co-localization of *ars*/*pho*/*pst*/*aio* genes (Supplementary Figure 1) is not unique to strain 5A, as it has been documented in many bacteria and referred to as an “arsenic island” (Li et al., 2013). This implies the likelihood of interrelatedness between arsenic and phosphorus metabolisms and that some level of regulatory synergy might be anticipated (Li et al., 2012, 2013). Our prior work concerning this island indicated that this occurs in strain 5A and the demonstrated connectivity in the current study now comprehensively confirms these prior observations and serves to validate the transcriptional changes observed throughout the current data set. In this context, we note that ArsR regulation only concerns the *pho/pst1* locus, which is up-regulated primarily by As(III), not the *pho/pst2* operon located elsewhere in the genome (Supplementary Figure 1) and should be regarded as the true phosphorus-stress response operon found and characterized in other Gram-negative bacteria (Hsieh and Wanner, 2010). We hasten to add that co-regulation between *ars*, *aio*, and *pho/pst1* does not support the claim (Wolfe-Simon et al., 2010) that arsenate can functionally replace phosphate in structures such as nucleic acids or ATP metabolism (Rosen et al., 2011 Bioessays).

While induction of As(III) oxidation is controlled by the PhoRB and AioXSR systems (Kang et al., 2012; Wang et al., 2018; Rawle et al., 2019) and requires phosphate-limiting conditions, ArsR-based regulation is not influenced by phosphate levels (nor redox conditions) and consequently the role(s) of the ArsRs in this organism can be separated from these other As(III)-sensitive regulatory systems. Hence, the high phosphorus media used here (12 mM) avoids complications resulting from the global nature of the PSR (Hsieh and Wanner, 2010; Rawle et al., 2019) as well as transcriptional controls expected from AioXSR (Kang et al., 2012; Wang et al., 2018; Rawle et al., 2019). At a very general level, some As(III)-affected gene functions in *A. tumefaciens* 5A were also observed in microarray studies of “early phase” *H. arsenicoxydans* described by Cleiss-Arnold et al. (2010), which were not initially exposed to PSR conditions (as opposed to the “late phase” cells, which were). However, there are also many differences observed between *H. arsenicoxydans* and strain 5A, which may derive from the increased sensitivity of RNASeq in the current study as compared to microarrays. Some examples include perturbation of many sugar transporters, opposite responses for copper tolerance, ± regulation of iron acquisition, and down-regulating molybdenum transport (Supplementary Table 2).

The structural genes for As(III) oxidase (*aioBA*) have thus far been observed to be co-expressed in wild-type organisms as a two-gene operon – as would be expected (e.g., Kashyap et al., 2006; Cai et al., 2009b). However, altered *aioB* expression in all four *arsR* mutants in the absence of As(III) was unexpected and suggests low-level ArsR-linked constitutive expression of *aioB* in the absence of As(III). At this juncture, it is unclear why/how these genes are differentially expressed. Studies describing *aioBA* expression in strain 5A have consistently shown the requirement of As(III) and to be under the control of AioXSR (Kang et al., 2012; Wang et al., 2018). Here, *aioB* transcription was observed under high P conditions, without *aioSRX* up-regulation, in the absence of As(III) (Table 1), excluded *aioA*, and with ArsR2 and ArsR4 apparently normally playing some type of activation role(s) when the cell is exposed to As(III) (Table 1 and Figure 3). At present, we are unable to suggest a mechanism to account for this apparent ArsR positive regulatory effect. Unlinked expression of *aioB* from *aioA* could potentially be explained by a repressor that binds directly downstream of *aioB* and that blocks transcription readthrough to *aioA* – but apparently only under high phosphate conditions. Clearly, significant work remains.

The inference of ArsR positive regulator of gene function is novel. The regulatory profiles of all ArsRs in strain 5A include various transcriptional regulators (Supplementary Tables 3–6), including genes annotated as activators and thus are likely involved in this context; i.e. de-repression of a transcriptional activator would lead to secondary level activation. These putative regulators might also be active as transcriptional modulators. An example of this can be seen when comparing As(III)-exposed wild type against the Δ*arsR4* mutant. Genes involved in chemotaxis and iron acquisition/homeostasis were down-regulated in the mutant (Table 1 and Supplementary Table 6), which would lead to the expectation that they are up-regulated in the wild-type cell containing ArsR4. However, the expected up-regulation appears modulated, by perhaps another regulator, such that there is no change in the wild-type cell at lower As(III) levels or indeed down-regulated at the highest As(III) level. The biological significance and cellular response logic is obscure even though the data demonstrating the possibility of it occurring is not.

One final point bears consideration. The novel observations reported in this study have relevance beyond soil or water environments where microbe–arsenic interactions have traditionally been studied. Initial work examining the impact of arsenic on the gut microbiome has clearly established that there are numerous and significant effects, but thus far the basis for these effects is unknown. An initial fate of ingested arsenic is to interact with the gastrointestinal tract microbiome wherein numerous transformations are possible (Coryell et al., 2018; McDermott et al., 2019) and that will have consequences for the host as well as other microbiome members (McDermott et al., 2019). Initial work examining the impact of arsenic on the gut microbiome have clearly established that there are numerous and significant effects, including altered microbiome structure and composition as well as metabolomic profiles (Lu et al., 2014; Chi et al., 2017, 2019a,b; Liu et al., 2019); however, thus far the basis for these changes is unknown. The *ars* genes/operons of Gram-positive and Gram-negative organisms are quite common in the human gut microbiome (McDermott et al., 2019), and there is no reason to view their transcriptional control to be any different to that shown in numerous pure culture isolates from virtually every other environment. Now, in addition to the specific *ars*-encoded functions, the very extensive profile of functions influenced by the ArsR protein documented herein offers a qualitative indication of what might be expected in the human gut microbiome and assists in explaining the very significant microbiome metabolic changes observed in different mouse studies (Lu et al., 2013; Guo et al., 2014; Lu et al., 2014; Richardson et al., 2018; Chi et al., 2019c; Liu et al., 2019).

Summarizing, the RNASeq experiments described herein illustrate that arsenic-stimulated ArsR regulation extends well beyond current paradigms of bacterial arsenic resistance (i.e. the *ars* operon model). Rather, ArsR regulation involves genes/operons representing an extraordinary array of cell functions (Figure 4), leading us to suggest that it is reasonable to conclude that the overall impact(s) of ArsR regulation will include altering community structure and function, not just resistance.

## Data Availability Statement

The datasets presented in this study can be found in online repositories. The names of the repository/repositories and accession number(s) can be found in the article/Supplementary Material.

## Author Contributions

TS conducted culturing, nucleic acid extraction, and some transcriptomics analyses, and provided edits. RR conducted the transcriptomics analyses and provided edits. Y-SK generated the mutants. QW conducted data analysis and editing. SW and BB provided intellectual input and manuscript editing. TM conceived the project, designed the study, and wrote the manuscript. All authors contributed to the article and approved the submitted version.

## Conflict of Interest

The authors declare that the research was conducted in the absence of any commercial or financial relationships that could be construed as a potential conflict of interest.
