# Supplementary figures and images for: Introducing the ArsR-Regulated Arsenic Stimulon
Source: Front Microbiol. 2021 Mar 3;12:630562. doi: 10.3389/fmicb.2021.630562 (PMC7965956; doi:10.3389/fmicb.2021.630562)

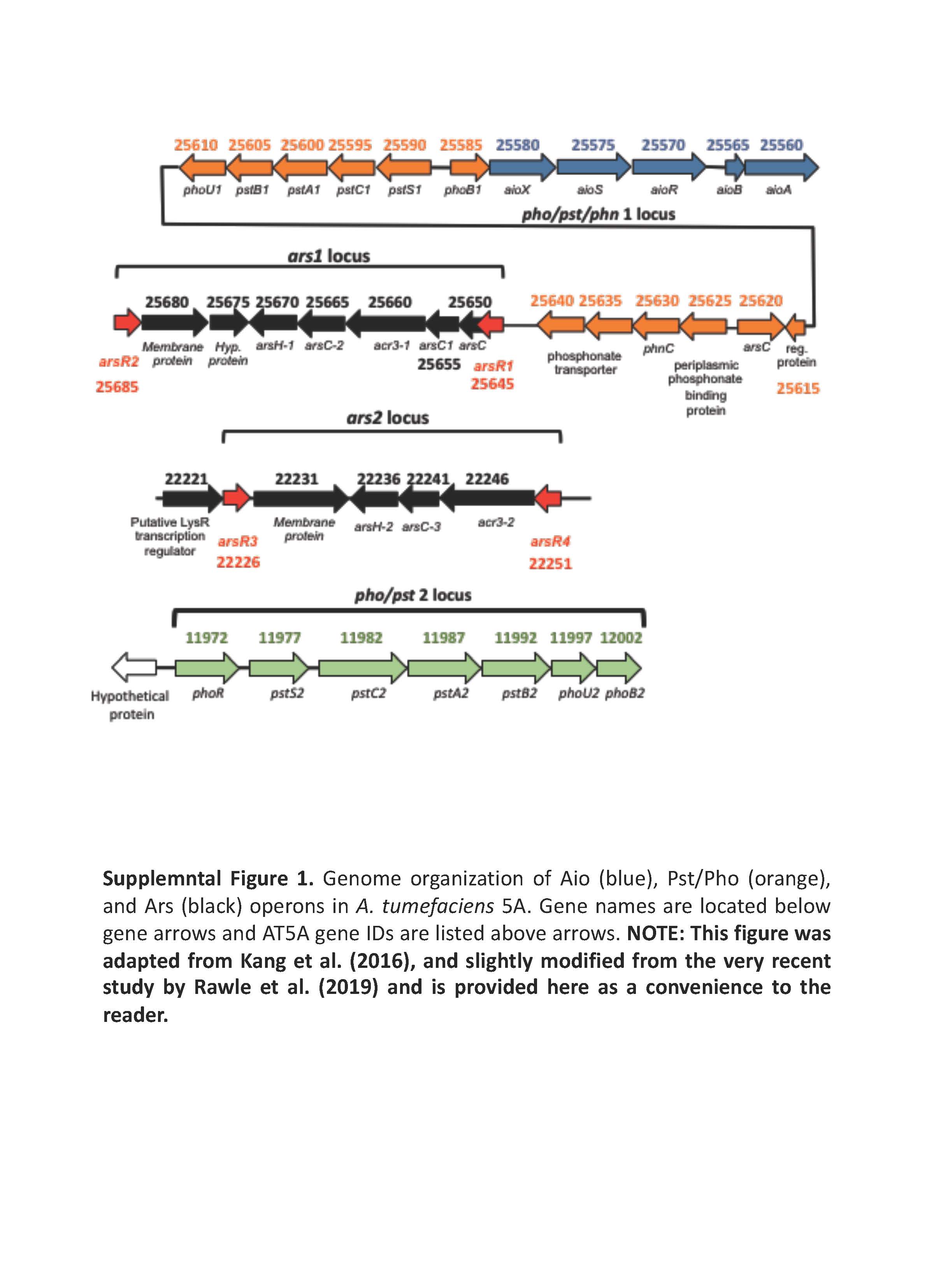

Supplement: Supplementary file 1 [file Image_1.JPEG]

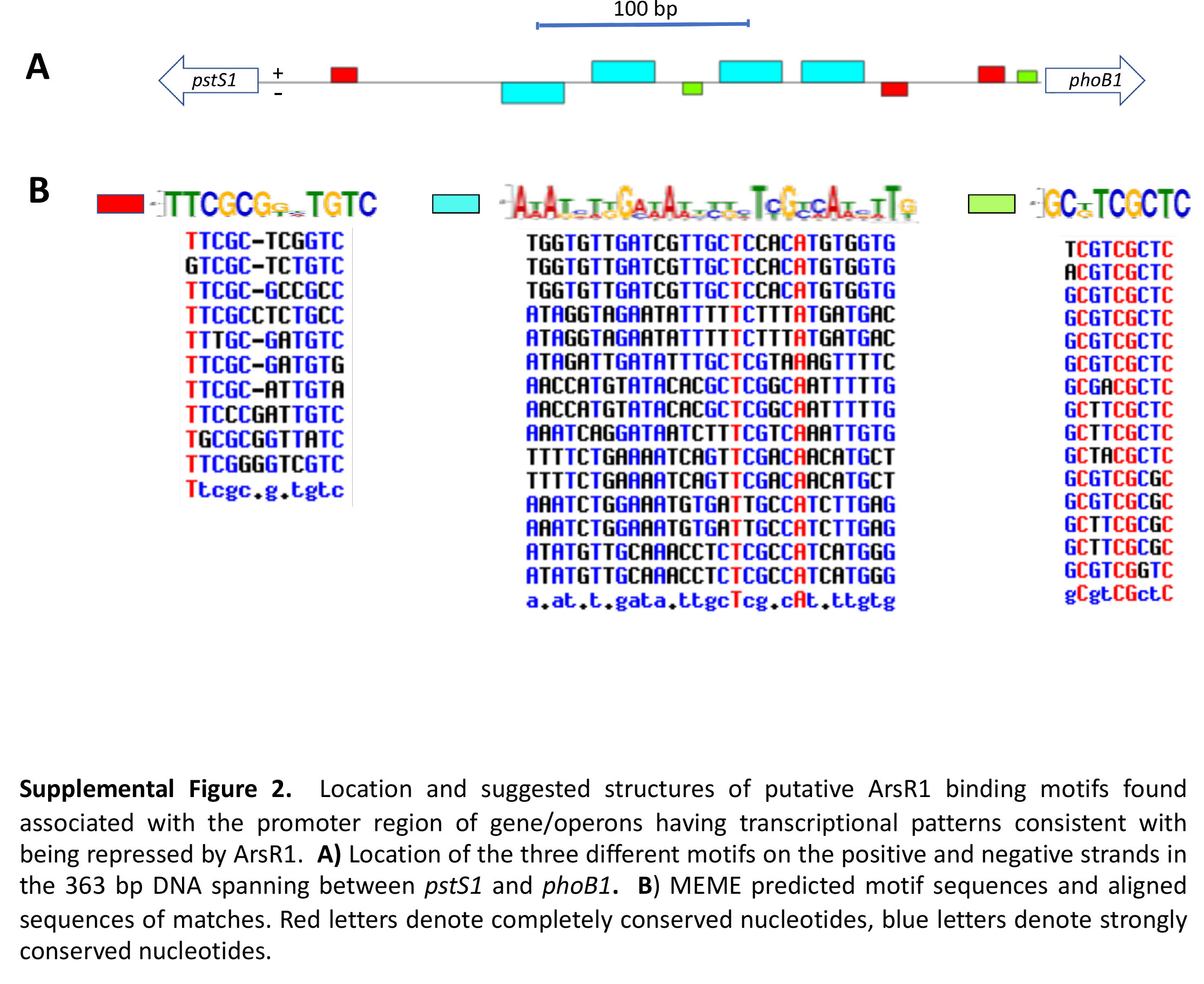

Supplement: Supplementary file 2 [file Image_2.JPEG]
