# Supplementary material for: Introducing the ArsR-Regulated Arsenic Stimulon
Source: Front Microbiol. 2021 Mar 3;12:630562. doi: 10.3389/fmicb.2021.630562 (PMC7965956; doi:10.3389/fmicb.2021.630562)
Supplement: Supplementary file 3 [file Table_1.pdf]

Suppl. TABLE 1. Bacterial strains and constructs used in this study.

Strains	Relevent markers and characteristics	Reference or Source
5A	Wild type, soil isolate, As(III) oxidizer	Lab stock
Δ*arsR1* mutant	*arsR1* gene deletion mutant	Kang et al. 2016
Δ*arsR2* mutant	*arsR2* gene deletion mutant	Kang et al. 2016
Δ*arsR3* mutant	*arsR3* gene deletion mutant	Kang et al. 2016
Δ*arsR4* mutant	*arsR4* gene deletion mutant	Kang et al. 2016
Constructs		
lacZ::*arsR1*	*ParsR1-lacZ fusion*	Kang et al. 2016
lacZ::*arsR2*	*ParsR2-lacZ fusion*	Kang et al. 2016
lacZ::*arsR3*	*ParsR3-lacZ fusion*	Kang et al. 2016
